# Study of Hexane Adsorption on Activated Carbons with Differences in Their Surface Chemistry

**DOI:** 10.3390/molecules23020476

**Published:** 2018-02-22

**Authors:** Diana Hernández-Monje, Liliana Giraldo, Juan Carlos Moreno-Piraján

**Affiliations:** 1 Departamento de Química, Facultad de Ciencias, Universidad Nacional de Colombia, Sede Bogotá, Carrera 30 No 45-03, Bogotá 111321, Colombia; dichernandezmo@unal.edu.co; 2 Departamento de Química, Facultad de Ciencias, Universidad Nacional de Colombia, Sede Bogotá, Carrera 30 No 45-03, Bogotá 111321, Colombia; lgiraldogu@unal.edu.co; 3 Departamento de Química, Facultad de Ciencias, Universidad de los Andes, Carrera 1 este No 18A-10, Bogotá 111321, Colombia

**Keywords:** activated carbon, hexane, gas phase isotherm, immersion calorimetry, energetic characterization, enthalpy of immersion, characteristic energy

## Abstract

The study of aliphatic compounds adsorption on activated carbon can be carried out from the energetic change involved in the interaction; the energy values can be determined from isotherms or by the immersion enthalpy. Vapor phase adsorption isotherms of hexane at 263 K on five activated carbons with different content of oxygenated groups and the immersion enthalpy of the activated carbons in hexane and water were determined in order to characterize the interactions in the solid–liquid system, and for calculating the hydrophobic factor of the activated carbons. The micropore volume and characteristic energy from adsorption isotherms of hexane, the BET (Brunauer–Emmett–Teller) surface area from the adsorption isotherms of N_2_, and the area accessible to the hexane from the immersion enthalpy were calculated. The activated carbon with the lowest content of oxygenated groups (0.30 µmolg^−1^) and the highest surface area (996 m^2^g^−1^) had the highest hexane adsorption value: 0.27 mmol g^−1^; the values for E_o_ were between 5650 and 6920 Jmol^−1^ and for ΔH_im_ were between −66.1 and −16.4 Jg^−1^. These determinations allow us to correlate energetic parameters with the surface area and the chemical modifications that were made to the solids, where the surface hydrophobic character of the activated carbon favors the interaction.

## 1. Introduction

Activated carbons are adsorbent solids that can develop different degrees of hydrophobicity, according to the oxygenated group content on the surface; this property favors the adsorption of organic compounds (aliphatic) of low molecular weight that may be present in the atmosphere increasing its levels of contamination [1]. In recent years, such compounds have been called volatile organic compounds (VOCs) and show high vapor pressure at room temperature, which allows them to easily reach the gas phase, in case of spills or leaks. VOCs are toxic and lethal to humans and cause environmental degradation [2].

As porous material, activated carbon has a high surface area and pore volume, which makes it a good adsorbent in both gas and liquid phase processes; another advantage is its versatility in fabrication, i.e., the possibility of modifying its porous structure and surface chemistry based on the requirements established in a specific application [3].

Due to the inert nature of the activated carbon surface, the affinity for low molecular weight molecules such as nitrogen or oxygen at room temperature or polar molecules such as water is very low. This very nature affords it a high affinity for non-polar molecules of a certain volume and molecular weight (e.g., hydrocarbons). This difference in affinity entails that activated carbon is a suitable adsorbent when gas phase separation or purification is performed [4].

There is much research reported in the literature about the adsorption of VOCs on activated carbons [5,6,7,8,9,10,11,12,13,14]. Among these compounds is hexane (C_6_H_14_), which is a non-polar compound of a linear aliphatic chain and establishes an interaction of a dispersive type with the adsorbent. Hexane partly constitutes gasoline and is used in the extraction of edible seed products and vegetable crops (e.g., soy, peanuts, and corn), as a cleaning agent (degreaser) in the printing industry, as a solvent in polymerization reactions, and in the development of certain adhesive products, lacquers, varnishes, inks, cements, and paints. It is also used as an alcohol denaturant and in thermometers for low temperatures, instead of mercury. Finally, in the laboratory, it is used as a solvent and as a raw material in synthesis.

However, its removal is relevant since acute and short-term exposure (by inhalation) affects the central nervous system (CNS) with such afflictions as dizziness, vertigo, nausea, headache, numbness of the feet and hands, and muscle weakness in the legs and feet. Continuous or chronic exposure is associated with dyschromatopsia, polyneuropathy in humans (sensitivity, muscular strength, and mobility affection), in turn, can cause paralysis of the arms and legs with numbness in the extremities, muscle weakness, blurred vision, headache, and fatigue [15,16,17,18,19].

The study of the adsorption of aliphatic compounds (such as hexane) on activated carbon can be carried out from the point of view of energetic change. This change involves interaction between the adsorbent and the adsorbate; the energy values can be determined by the gas phase isotherms or by the energy released when the compound in liquid phase contacts the solid [20].

The enthalpy of immersion allows one to determine, depending on the thermodynamic conditions of the system, the energy transfer that occurs when a solid and a liquid are brought into contact with each other. The thermal effects resulting from submerging a solid in a solvent, generally of a non-polar type, with which the solid does not present chemical interactions, are related to the surface area of the considered solid through the models developed by Dubinin and Stoeckli [21].

For an activated carbon with a micropore volume, W_o_, a characteristic energy, E_o_, and an adsorbate in liquid phase with molar volume, V_m_, Stoeckli and Kraehenbüehl [22] proposed the equation
(1)
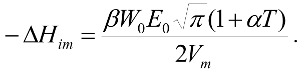


The relationship between the enthalpy of immersion and the micropore volume, which indicates that enthalpy corresponds to the micropore filling process and to the wetting of open or non-porous surfaces, is shown.

The experimental enthalpy of immersion, ΔH_exp_, of an activated carbon contains two types of contributions: one due to the interaction of the adsorbate with micropores and the other to the wetting of the external surface, S_ext_, as described by Stoeckli, Bansal, and Donnet [23]. This can be expressed by
(2)
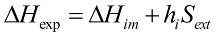

where *h_i_* represents the specific enthalpy, which is determined with different immersion liquids for solids that do not have porosity [24].

From the above equation, the external surface can be calculated once the specific enthalpy, *h_i_*, is known:
(3)
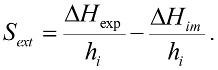


This equation shows that the determination of the enthalpy of immersion of an activated carbon in a non-polar liquid solvent allows one to calculate the total area of the solid; when the activated carbon has no external surface or development of meso- and macropores, the experimental enthalpy, ΔH_exp_, is similar to the enthalpy, ΔH_im_, calculated with the Stoeckli–Kraehenbüehl equation.

Thus, it is interesting to quantify not only how the adsorbed amount of hexane is affected by differences in the surface of a series of activated carbons, but also what change in the solid–adsorbate interaction energy determines its adsorption. Other authors have studied hexane adsorption in porous materials, such as activated carbons, activated carbon cloths, and activated carbon fiber cloths, using solids with different precursors and different preparation and modification methods and generating diverse chemical and textural properties, and found areas between 502 and 1565 m^2^g^−1^ and adsorption capacities between 1.030 and 5.730 mmolg^−1^. 

These adsorption capacities have higher values than those reported in this work, possibly due to the fact that the samples used in the previously reported investigations are highly microporous; in addition, the adsorption occurred at higher temperatures, increasing the value of the saturation pressure, which is proportional to the number of moles injected from the gas phase, increasing the amount of adsorption [25,26,27,28,29,30,31,32].

With regard to immersion enthalpy values, the values shown in this work (between −16.4 and −66.1 Jg^−1^) are consistent with previously reported values, these are between −25.23 and −127.8 Jg^−1^ for highly microporous activated carbons, with areas between 752 and 1208 m^2^g^−1^ [33,34].

In this work, the hexane adsorption isotherms on activated carbons with different surface physicochemical properties are determined, and the enthalpies of the immersion of the solids in hexane and water are calculated. This is done to establish the difference between the micropore volume and surface area values obtained by the isotherms of the adsorption of both N_2_ and hexane, and to establish the relationships between the enthalpy of immersion, the surface area of the activated carbon, and the characteristic energy of adsorption obtained from the adsorption isotherms of hexane and the immersion calorimetries of the modified solids in hexane.

## 2. Results and Discussion

### 2.1. Relationship between the Physicochemical Characteristics of Activated Carbons and Gas Phase Adsorption Isotherms of Hexane

Table 1 presents physicochemical characteristics of the activated carbons of this study, including the surface area obtained by the BET (Brunauer–Emmett–Teller) model, the micropore volume calculated by the DR (Dubinin-Radushkevich) model, the average size pore determined by the QSDFT (Quenched Solid State Functional Theory model, based on the adsorption isotherms of N_2_ and the total oxygenated functional group content on the surface [35]. It can be seen that the apparent surface area values increase proportionally with the thermal treatment carried out on the samples; however, the micropore volume values are quite similar, which may be due to two processes, oxidation and subsequent thermal treatment: The first is the decrease in microporosity due to oxidation and the subsequent increase due to the increase in temperature, generating opposite effects; if this occurs, microporosity values similar to the starting sample will be obtained. The second is the widening of supermicropores, which would give rise to greater mesoporosity; this would increase the surface area but would not significantly increase the volume of micropore. Besides, a higher mesoporosity can be due to a greater amount of oxygen functional groups that generates more active adsorption sites [36].

On the other hand, when the content of oxygenated groups is obtained by Boehm titrations, the groups determined correspond to lactonic, carboxylic, and phenolic groups present in the samples. When the heat treatment was performed, a decrease in the amount of oxygenated groups was evidenced as the activation temperature increased because carboxylic groups decompose between 523 and 673 K, lactones between 673 and 923 K, and phenols between 873 and 1073 K, and the quinone and pyrone groups decompose at temperatures above 1173 K [37,38]. From there, activated carbon at 1173 K shows the lowest amount of carboxylic, lactone and phenol groups. In the modification with nitric acid, an increase in the presence of evaluated oxygenated groups was observed, which was due to the oxidation produced by the HNO_3_, which leads to the formation of acidic groups on the activated carbon surface, favoring mainly the formation of carboxylic groups [39].

**Figure 1:**
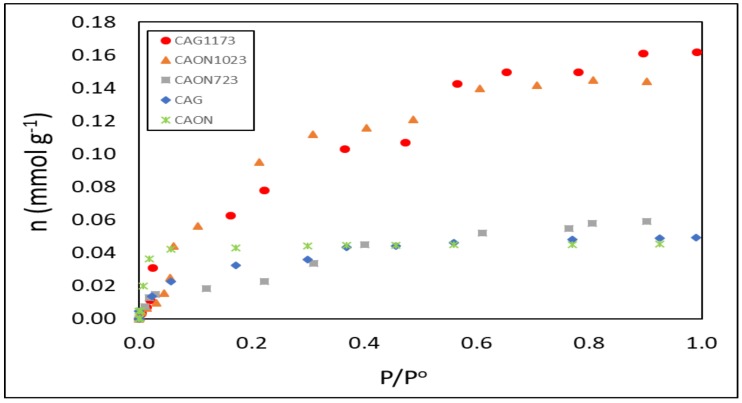
Adsorption isotherms of hexane on the five activated carbons at 263 K.

Figure 1 shows the adsorption isotherms of hexane at 263 K for the five activated carbons of this study. The highest values of millimoles of hexane adsorbed were the ones of the activated carbons exposed to thermal activation, and these quantities increased when the temperature of activation increased. The adsorption data have an uncertainty of 1 × 10^−4^ on the scale of mmolg^−1^, which allows one to distinguish between adsorption values in the same isotherm and shows differences in the adsorption of the activated carbons that are studied; for example, for CAG and CAON for relative pressures greater than 0.7, the adsorption values of each point are different, although those are low values.

Later, the isotherms were fitted to the Langmuir and Freundlich models. Langmuir model parameters were determined, such as the monolayer adsorption capacity (n_m_), the Langmuir constant (b), and the correlation coefficient of the plots. According to the Freundlich model, the constant value, the 1/n parameter, and the correlation coefficient were calculated. The data are shown in Table 2.

The adsorption isotherms are adjusted to the two models. Regarding the parameter 1/n, there are heterogeneous structure for all samples (all values are less than 1), and the intensity of interactions between the solid and the hexane increases proportionally with the temperature of the heat treatment. On the other hand, K_f_ and n_m_ are related to adsorption capacity. The results corroborate the influence of thermal changes on the adsorption process, since these changes increase the adsorption capacity of hexane even in the samples that were oxidized [40,41]. Concerning the Langmuir model, CAG1173 showed the highest adsorption capacity (0.27 mmolg^−1^), evidencing a fivefold increase with respect to the adsorption of the starting activated carbon CAG (0.05 mmolg^−1^), followed by the solid CAON1023 (0.19 mmolg^−1^) and finally by the activated carbon CAON723 being treated at a lower temperature (0.09 mmolg^−1^).

In the adsorption of hexane for the activated carbon treated with a nitric acid solution, a decrease and the lowest value for the series of the five solids was evidenced. The heat treatment at temperatures between 723 and 1173 K favored the area available for the adsorption of hexane molecules on the solid. The chemical modification restricted the path of the molecules to the porous network.

The increase in the temperature caused the removal of surface groups. At temperatures near 723 K, carboxylic groups of the surface were removed, and, at 1023 K, some anhydrides and lactones were decomposed. At 1173 K, the groups mentioned above were removed, as were carbonyls, phenolics, ethereal groups, and some quinones. Treatment with the HNO_3_ solution generated a greater amount of oxygenated surface groups, such as carboxylic, lactonic, and phenolic groups, which made the entrance of the hexane to the porous network difficult and even generated inaccessibility to certain regions of the solid in the edges of the graphene layers [42,43,44,45].

### 2.2. Relationship between the Physicochemical Characteristics of Activated Carbons and the Enthalpy of Immersion

**Figure 2:**
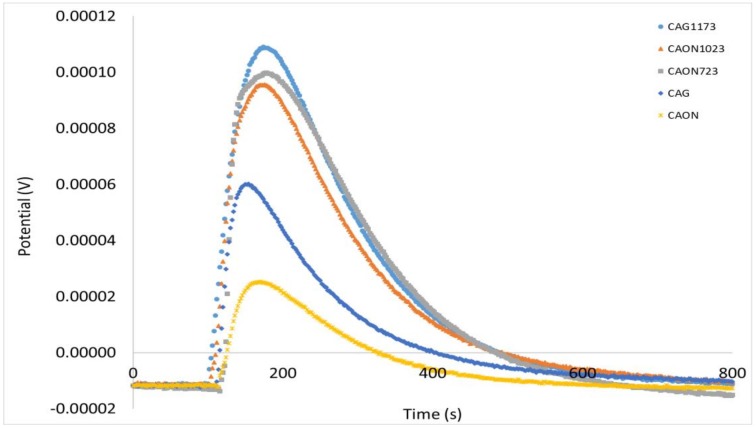
Calorimetry curves of the immersion of the activated carbon into hexane.

Figure 2 shows the plot of electrical potential as a function of time, obtained for the immersion of the activated carbons into hexane. These data allows one to calculate the enthalpy of immersion. The immersion of solids was also performed in water. The values derived from calorimetric determinations were performed in triplicateand the standard deviation isbetween 0.33 and 2.65 Jg^−1^. Based on the enthalpy of immersion in hexane and water, the hydrophobic factor of the solids was calculated with respect to hexane [46]. The enthalpy of immersion of hexane and water, as well as the hydrophobic factor are shown in Table 3.

The area of the peak under the potential curve as a function of time is proportional to the amount of heat generated in the solid–liquid contact. It was observed that the activated carbon with the highest peak in the hexane immersion is the one that was heat-treated at 1173 K and had the lowest oxygenated group content at the surface, showing that the decrease of oxygenated groups on the surface increased the enthalpy of immersion of the solid in the aliphatic solvent [46]. The effect that the immersion produces increases the potential of the thermal sensor, indicating that the process is exothermic and involves interactions between the components of the solid–liquid system being studied.

The values of the enthalpy of immersion of the activated carbons in water were also exothermic, but of a smaller magnitude for the solids that were heat-treated; for the CAG and CAON, the values of the enthalpy of immersion in water are higher than those in hexane.

**Figure 3:**
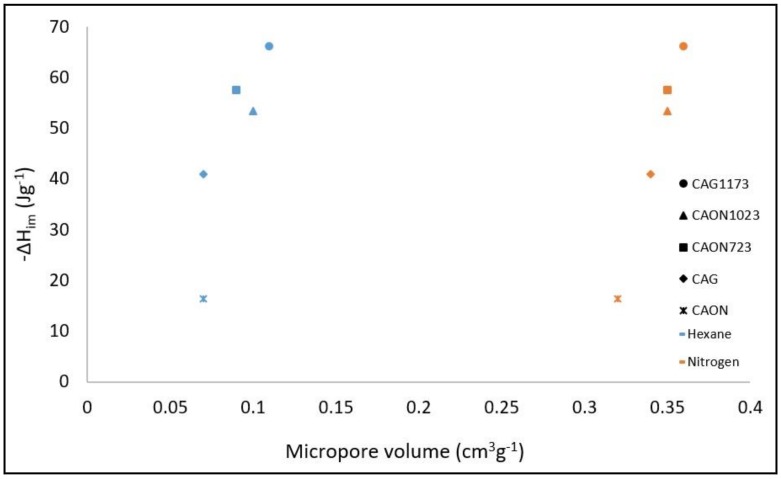
Immersion enthalpy of the activated carbons in hexane as a function of the micropore volume.

A relationship between the enthalpy of immersion of the activated carbon in hexane and the micropore volume calculated by the Dubinin–Radushkevich model was established for both N_2_ and hexane isotherms. The results are presented in Figure 3. In Table 4, the area accessible to hexane, the characteristic energy of adsorption (E_o_), and the micropore volume (V_o_) calculated by the DR model from the adsorption isotherms of C_6_H_14_ are shown.

The values for the micropore volume were lower when they were calculated from the hexane isotherms compared with when they were determined for the N_2_ isotherms, because the hydrocarbon molecule has a larger size than nitrogen; therefore, the capacity to occupy the pores that are classified in the range of micropores is smaller.

It was observed in both cases that, as the volume of micropores increased, the value of the enthalpy of immersion increased too, which reflects the interaction of hexane with the surface of the solid [22].

**Figure 4:**
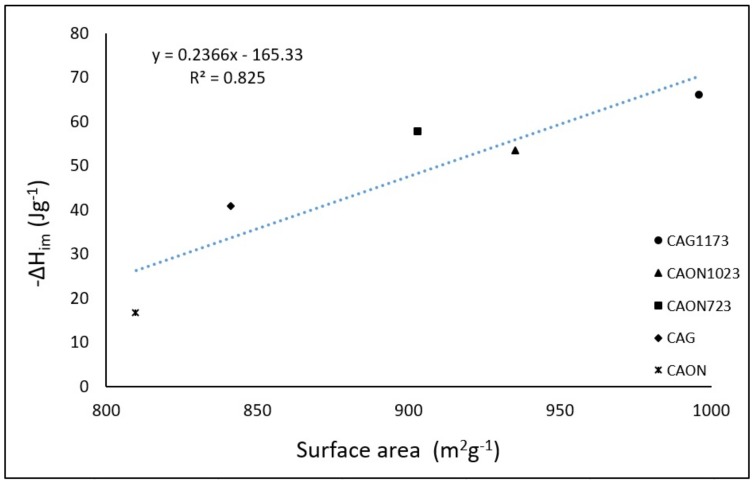
Immersion enthalpy of activated carbons in hexane as a function of the surface area.

As is known, the enthalpy of immersion of a solid into a liquid that produces dispersive interactions (solid–liquid contact) is proportional to the surface area of the solid [21]. The relationship between the surface area of the activated carbon and the enthalpy of immersion in hexane is presented in Figure 4.

The proportionality between the two parameters exists because there is a larger space available for the entrance of the molecules and this generates a greater interaction between the adsorbate and the porous structure.

An activated carbon with a higher specific surface area and a greater interaction was CAG1173, which presented a higher surface area due to the selective removal of oxygenated groups and produced higher values of enthalpy of immersion because of the affinity between the adsorbate and the carbonaceous structure.

The activated carbon CAON had the lowest surface area value and the lowest enthalpic interaction because the surface oxygenated groups decreased the available space of the activated carbon for the hexane entry and generated less interaction with the molecules because of its polar character.

### 2.3. Relationship between the Energetic Characterization (Characteristic Energy and Enthalpy of Immersion) and Physicochemical Characteristics of Activated Carbons

**Figure 5:**
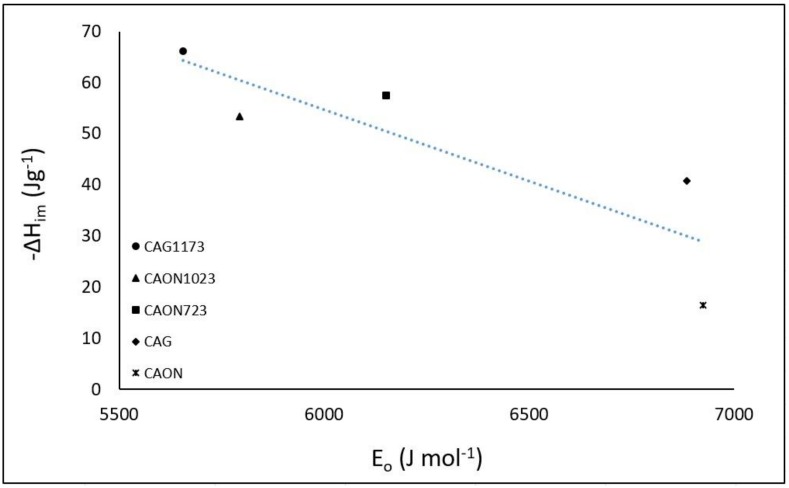
Enthalpy of immersion enthalpy of the activated carbons in hexane, ΔH_im_, as a function of the characteristic energy of adsorption, E_o_.

The characteristic energy of adsorption, E_o_, for the activated carbons from the hexane adsorption isotherms was calculated (Table 4). This is an energy parameter typical of each adsorbate–adsorbent system. Figure 5 shows the relationship between the enthalpy of immersion of the activated carbon in hexane and the characteristic energy of hexane adsorption on the solids, which are the two energetic characteristics that are calculated in this study.

Figure 5 shows the energetic property, ΔH_im_, determined in liquid phase, and another energetic property, E_o_, determined in vapor phase. Increasing the characteristic energy of adsorption decreased the enthalpy of immersion, so the hexane adsorption process in the set of activated carbons was favored when the enthalpy of immersion values were higher and the characteristic energy of adsorption was lower.

**Figure 6:**
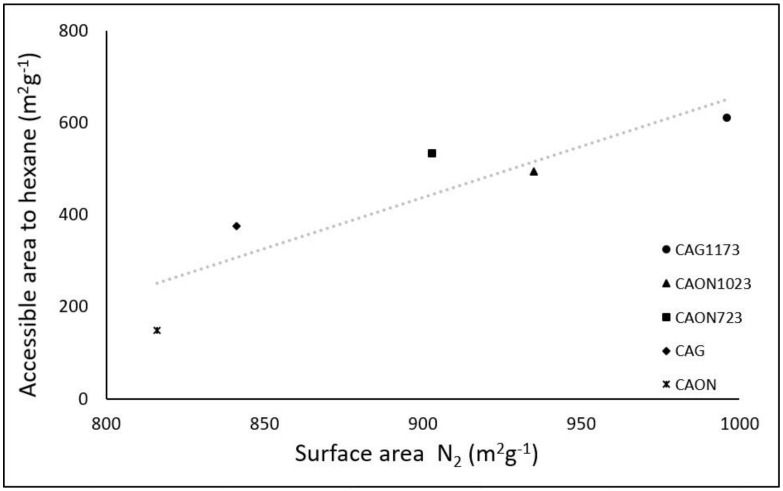
Area accessible to hexane as a function of BET surface area.

As mentioned in the introduction, based on the enthalpy of immersion of the activated carbon in hexane, the area accessible to hexane can be calculated by knowing the surface enthalpy value [47], which for hexane is −0.108 Jm^2^. If the surface area calculated from the N_2_ adsorption isotherm via the BET model and the area accessible to hexane are known, a relationship can be established, as shown in Figure 6.

The values of the BET surface area were higher than those obtained for the area accessible to hexane due to the difference in the size of the two molecules, but a direct proportional relationship between the two values was observed. This result confirms that the molecule of each adsorbate occupies a certain space on the surface of the solid. Depending on the molecular size, there will be sites of the solid that larger molecules cannot access. This is consistent with values found for the average pore width, which is between 0.753 and 0.785 nm according to the QSDFT model (Table 1), showing that the nitrogen molecule with a molecular size of 0.29 nm can enter the porous structure more easily compared to the hexane molecule, whose molecular size is 0.67 nm, quite close to the average pore width value determined for the samples.

Finally, the hydrophobic factor of the activated carbons with respect to the hexane was calculated. The hydrophobic factor, *h_f_*, is a relationship between the enthalpy of immersion of the activated carbons in hexane and the enthalpy of immersion in water and allows one to establish a scale of hydrophobicity of the surface of the solid [48].

**Figure 7:**
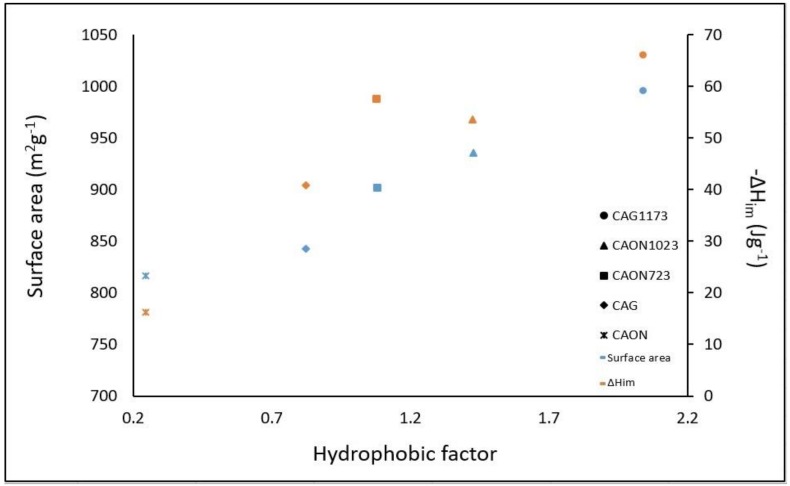
Relationship between the hydrophobic factor, the BET surface area, and the enthalpy of immersion of the activated carbons in hexane.

The hydrophobic factor was related to the surface area and the enthalpy of immersion of the activated carbons in hexane, and the results are presented in Figure 7.

It was observed that, as the hydrophobic factor increased, both the BET surface area and the enthalpy of immersion in hexane increased. Thus, the interaction of hexane with the surface of the solids increased when the content of surface groups diminished and thus predominated the hydrophobic character on the surface such that the interaction with hexane was greater. The BET surface area and the enthalpy of immersion are directly proportional properties and they are affected by the content of oxygenated groups on the surface.

## 3. Materials and Methods

### 3.1. Activated Carbon

The modification of a starting activated carbon prepared from coconut shell by physical activation (CAG) was carried out. It was sieved to a particle size of 1 mm and was washed with distilled water, then dried for 24 h at 363 K and stored in containers under a nitrogen atmosphere.

Two types of treatment were carried out on CAG: a chemical modification of oxidation with HNO_3_ 6 M solution for the development of oxygenated surface groups (CAON) and a thermal treatment in an inert atmosphere (N_2_), allowing for the selective decomposition of superficial groups, giving rise to five samples. The denomination of the activated carbons and the chemical and thermal treatments to which the activated carbons were subjected are shown in Table 5.

### 3.2. Nitrogen Adsorption Isotherms

The nitrogen adsorption isotherms were performed at 77 K in an Autosorb 3B, Quantachrome equipment to determine the apparent surface area and the micropore volume using the BET and Dubinin–Radushkevich models, respectively.

### 3.3. Boehm Titrations

Boehm titrations are used to determine the oxygenated group content (lactonic, phenolic, and carboxylic surface groups) of activated carbons: 0.5 g of activated carbon were weighed and mixed with 50 mL of NaOH, Na_2_CO_3_, NaHCO_3_, and HCl 0.1 M solutions. Subsequently, they were kept under stirring and at a constant temperature of 293 K for five days. Finally, an aliquot of 10 mL of each of the solutions in contact with the solid was taken and titrated with previously standardized HCl (NaOH, Na_2_CO_3_, NaHCO_3_) or NaOH (HCl) [49,50]. The titrations were carried out using a CG 840B Schott potentiometer.

### 3.4. Hexane Adsorption Isotherms

The gas-phase adsorption isotherms of hexane (Merck-brand analytical reagent) on the activated carbons were determined in a sortometer assembled in the laboratory. The equipment has a cell for the adsorptive with a capacity of 5.0 mL. The hexane moves through a stainless steel pipe and is stored in a tank. The temperature is regulated to 343 K to reach the gas phase, after which it is displaced to a glass cell where the activated carbon is located with a fixed isotherm temperature (263 K). The adsorption process is controlled by five valves that regulate the pressure of the system, which is measured by means of a digital sensor. The system has a Pfeiffer vacuum pump and an Edwards diffuser pump, which allows for a minimum pressure of approximately 3 × 10^−2^ mbar. The temperature of the system is also controlled by a Cole Parmer thermostat (263 K). Data collection is carried out through a digital multimeter and Extech software that captures the change in electric potential as a function of time. The electric potential is converted to pressure to obtain one point of the isotherm.

### 3.5. Enthalpy of Immersion of Activated Carbon in Solvents

Activated carbons were immersed in hexane and water to determine the enthalpy of immersion and to quantify the hydrophobic/hydrophilic character of the solids. To determine the enthalpy of immersion, 10 mL of the solvent were placed in a stainless steel cell assembled to the main heat reservoir of the calorimeter at 298 K; then, 100 mg of each activated carbon were weighed and placed in a glass vial fitted to the calorimeter cell designed for this purpose. Next, the system was left to sit for approximately 1 h, until the temperature of the calorimetric assembly stabilized or or the time required for stabilization of the calorimeter; later, the immersion of the sample into the liquid was performed, and the resulting thermal changes were recorded until the baseline was attained again. Finally, a post-period of 20 min was recorded and the experience of the electric calibration was executed [51].

## 4. Conclusions

The determination of the hexane adsorption isotherm on a series of activated carbons and the enthalpy of immersion of these in hexane allowed us to determine two energetic parameters—the characteristic energy of adsorption, E_o_, and the enthalpy of immersion, ΔH_im_—that characterize the activated carbon–hexane system, with values for E_o_ between 5650 and 6920 Jmol^−1^ and for ΔH_im_ between −66.1 and −16.4 Jg^−1^.

The micropore volume values for N_2_ and hexane adsorption were calculated. The values of the micropore volume for the hexane adsorption are lower because it has a larger molecular size.

It was observed that the hydrophobic character of the surface of the activated carbon favors the interaction with the hexane.

## Figures and Tables

**Table 1:** Physicochemical characteristics of activated carbons.

Activated Carbon	BET Surface Area (m^2^g^−1^)	V_o_(N_2_) (cm^3^g^−1^)	Average Pore Size (N_2_-QSDFT) (nm)	Oxygenated Group Content (µmolg^−1^)
CAG	840	0.34	0.785	0.33
CAON	816	0.32	0.753	0.54
CAON723	903	0.35	0.785	0.43
CAON1023	935	0.35	0.785	0.34
CAG1173	996	0.36	0.785	0.30

**Table 2:** Parameters obtained from the fit to the Langmuir and Freundlich models for the hexane isotherms on the modified activated carbons.

Activated Carbon	Langmuir			Freundlich		
	b (mbar^−1^)	n_m_ (mmolg^−1^)	R^2^	K_f_ (mmolg^−1^mbar^−1^)	1/n	R^2^
CAG	0.087	0.052	0.986	0.053	0.309	0.974
CAON	0.010	0.046	0.972	0.052	0.194	0.843
CAON723	0.463	0.090	0.948	0.064	0.503	0.973
CAON1023	0.261	0.193	0.989	0.171	0.520	0.959
CAG1173	0.627	0.269	0.983	0.172	0.589	0.984

**Table 3:** Parameters determined from the interaction between hexane and water with the activated carbon by immersion calorimetry.

Activated Carbon	−∆H_im_(C_6_H_14_) (Jg^−1^)	−∆H_im_(H_2_O) (Jg^−1^)	Hydrophobic Factor
CAG	40.9	49.6	0.07
CAON	16.4	66.6	0.07
CAON723	57.6	53.3	0.09
CAON1023	53.4	37.4	0.10
CAG1173	66.1	32.4	0.11

**Table 4:** Parameters determined from the adsorption isotherms of hexane (C_6_H_14_) by the DR (Dubinin-Radushkevich) model.

Activated Carbon	Area Accessible to Hexane (m^2^g^−1^)	E_o_(C_6_H_14_) (Jmol^-1^)	V_o_(C_6_H_14_) (cm^3^g^−1^)
CAG	379	6881	0.07
CAON	152	6922	0.07
CAON723	533	6149	0.09
CAON1023	494	5792	0.10
CAG1173	612	5656	0.11

**Table 5:** Activated carbons used in hexane adsorption.

Activated Carbon	Treatment
CAG	Starting carbon
CAON	CAG exposed to oxidation with a solution of HNO_3_
CAON723	CAON exposed to heat treatment at 723 K for 2 h under N_2_ atmosphere
CAON1023	CAON exposed to heat treatment at 1023 K for 2 h under N_2_ atmosphere
CAG1173	CAG exposed to heat treatment at 1173 K for 2 h under N_2_ atmosphere
